# Role of platelet to albumin ratio for predicting persistent acute kidney injury in patients admitted to the intensive care unit

**DOI:** 10.1186/s12871-023-02137-6

**Published:** 2023-07-19

**Authors:** Yuanwei Zhai, Xiaoqiang Liu, Yu Li, Qionghua Hu, Zhengwei Zhang, Tianyang Hu

**Affiliations:** 1 Department of Medical Imaging, the First People’s Hospital of Ziyang, Ziyang, Sichuan China; 2 Department of Orthopedic Surgery, Anyue County People’s Hospital, Ziyang, Sichuan China; 3 Department of Nephrology, Bishan Hospital Affiliated to Chongqing Medical University, Chongqing, China; 4 Department of Critical Care Medicine, Chengdu Second People’s Hospital, 10 Qingyunnan Street, Jinjiang District, Chengdu, 610017 Sichuan China; 5 Precision Medicine Center, the Second Affiliated Hospital, Chongqing Medical University, 74 Linjiang Road, Yuzhong District, Chongqing, 400010 China

**Keywords:** Platelet to albumin ratio, Persistent acute kidney injury, Intensive care unit

## Abstract

**Background:**

The aim of this study was to investigate the prognostic role of platelet to albumin ratio (PAR) and in persistent acute kidney injury (pAKI) of patients admitted to the intensive care unit (ICU).

**Methods:**

We involved pAKI patients from the Medical Information Mart for Intensive Care-IV (MIMIC-IV) database and eICU Collaborative Research Database (eICU-CRD). Receiver operating curve (ROC) analysis was performed to evaluate the optimal cut-off PAR.

**Results:**

A total of 7,646 patients were finally included in the present study. The optimal cut-off value of PAR was 7.2. The high-PAR group was associated with pAKI (hazard ratio [HR]: 3.25, 95% CI: 2.85–3.72, *P* < 0.001). We also performed this in the validation cohort, the results further confirmed that the high-PAR group was associated with pAKI (HR: 2.24, 95% CI: 1.86–2.71, *P* < 0.001). The PAR exhibited good pAKI predictive abilities in the original cohort (C-index: 0.726, 95%CI: 0.714–0.739) and in the validation cohort (C-index: 0.744, 95%CI:0.722–0.766) Moreover, as a systemic inflammatory indicator, PAR depicted better predictive ability compared to other systemic inflammatory indicators.

**Conclusion:**

The present study manifested that elevated PAR could predicts pAKI in patients admitted to ICU. PAR may be an easily obtained and useful biomarker to clinicians for the early identification of pAKI.

**Supplementary Information:**

The online version contains supplementary material available at 10.1186/s12871-023-02137-6.

## Introduction

Acute kidney injury (AKI) is commonly occurs with a high incidence which represents a global public health problem in patients admitted to the intensive care unit (ICU) and is associated with significant morbidity and mortality [1–3]. Reported mortality in ICU patients with AKI accounts for approximately 36–67% depending on AKI definition [2, 4]. Although efforts have been made to curb AKI progress to chronic kidney disease (CKD) and end-stage kidney disease (ESKD), there remains a considerable proportion of patients presenting to ICU who required renal replacement therapy (RRT) [5, 6]. Moreover, the AKI occurrence in ICU increases the length of stay, the need for more vasopressors drugs, and increased the cost of services and health care systems [7, 8].

Since the 2017 Acute Disease Quality Initiative (ADQI) workgroup proposed standard definitions of transient and persistent AKI (pAKI) based on the potential impact of AKI duration on outcomes [9], numerous investigators explored the outcomes of different types of AKI. Previous evidence indicated that two-thirds of patients with AKI resolve their renal dysfunction rapidly and there still almost one-third of patients progress to pAKI. pAKI patients exhibited an increased risk of CKD, ESKD, prone to receive RRT, and reduced survival compared to those transient AKI patients [10, 11]. Considering the important role of pAKI in the prognosis of critically ill patients, early and accurate risk assessment is of critical importance for clinical management in ICU patients to receive early interventions.

Clinicians are seeking clinically meaningful predictors or biomarkers for pAKI in ICU patients. A recent study intended to assess novel candidate biomarkers to predict pAKI in critically ill patients and found that urinary C-C motif chemokine ligand 14 (CCL14) is a predictive biomarker for pAKI in critically ill patients [12]. Shen et al. reported that 24-h procalcitonin change is a good predictor of pAKI in critical patients [13]. However, these biomarkers are not easily obtained upon admission to clinical. A simple and easily accessible prognostic biomarker for early risk stratification of pAKI in patients admitted to ICU is needed.

Platelet to albumin ratio (PAR) is a widely used biomarker clinically based on routine laboratory tests which reflect the systemic inflammatory state and nutrition status, has been reported to predict several disease settings [14, 15]. However, limited data have been presented on the relationship between PAR and pAKI in critically ill patients. This study sought to investigate the role of PAR in predicting pAKI in patients admitted to ICU.

## Methods

### Data source

All data were extracted from the eICU Collaborative Research Database (eICU-CRD, Additional file 1) [16] and the Medical Information Mart for Intensive Care-IV (MIMIC-IV version 1.0, Additional file 2) database [17]. This project was both approved by Beth Israel Deaconess Medical Center (BIDMC) and the institutional review boards of Massachusetts Institute of Technology (MIT) (certification number: 9,322,422). All procedures were performed according to the ethical standards of the Helsinki Declaration and its later amendments or comparable ethical standards. After finishing the web-based training courses and the Protecting Human Research Participants examination, we obtained permission to extract data from the eICU-CRD and MIMIC-IV databases.

### Patient and public involvement

Patients and/or the public were not directly involved in this study.

### Cohort selection

**Fig. 1:**
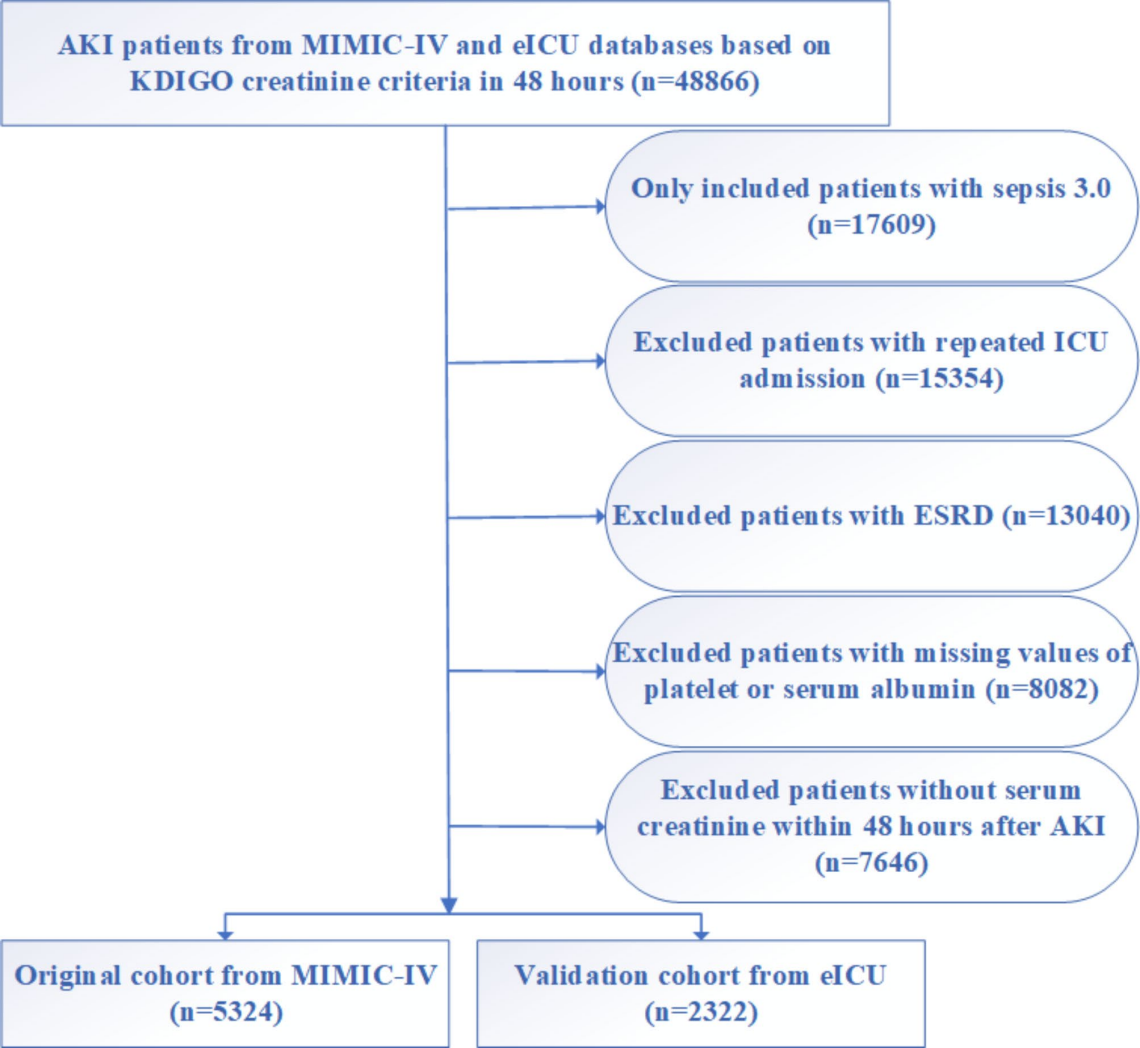
The flow chart of this study

The inclusion criteria in this study were as follows: (1) sepsis 3.0 criteria; (2) KDIGO-AKI criteria based on serum creatinine in the first 48 h of their ICU.

admission [18]. Patients with one of the following conditions were excluded: (1) less than 18-year-old at first admission to ICU; (3) more than 10% of personal data was missing; (4) patients with repeated ICU admissions; (5) patients without serum creatinine measures between 48 and 72 h after the diagnosis of AKI. A total of 5,324 patients in the MIMIC-IV database assigned to the original cohort and 2,322 patients in the eICU database assigned to the validation cohort were finally included in this study (Fig. 1).

### Data collection and outcomes

Baseline characteristics and admission information: age, gender, weight, ethnicity, and severity score measured by the sequential organ failure assessment (SOFA) score, the oxford acute severity of illness score (OASIS), the simplified acute physiology score II (SAPS II) were calculated as described in previous studies [19, 20]. Vital signs, comorbidities, laboratory indicators, Use of mechanical ventilation (MV) and renal replacement therapy (RRT) on the first day of their ICU admission were also recorded in this study. In addition, the use of drugs were also included in the present study. Moreover, initial vital signs and laboratory results were also measured during the first 24 h of ICU admission. Acute kidney injury (AKI) and persistent AKI (pAKI) were also extracted.

The primary outcome was pAKI.

### Definitions

Baseline creatinine was the minimum value on the first day of their hospital admissions. Recovery of AKI was defined as greater than or equal to a 50% decrease in serum creatinine after the diagnosis of AKI and/or return of serum creatinine to the baseline value. pAKI was defined as renal dysfunction without recovery within 2 days or before death [9]. PAR was defined as platelet to serum albumin ratio, NLR was defined as the neutrophil-lymphocyte ratio, PLR was defined as the platelet-lymphocyte ratio, and MLR was defined as monocyte-lymphocyte ratio counts. Systemic immune-inflammatory index (SII) is defined as platelet*neutrophil/lymphocyte.

### Statistical analysis

For all continuous covariates, the mean values and standard deviations are reported. Categorical data were expressed as frequency (percentage). The Chi-square test or Fisher’s test was appropriately performed to compare the differences between groups. The baseline characteristics were reported as an original cohort, matched cohort, and validation cohort. The receiver operating curve (ROC) analyses was conducted to evaluate the optimal cut-off PAR based on the Youden index, the cohort was then divided into two groups: the low-PAR group and high-PAR group.

Finally, the performance of NLR, PLR, MLR, and SII was assessed by the receiver operating curve (ROC) analyses. All analyses were performed in R software (version 4.1.0). *P* < 0.05 was considered statistically significant.

## Results

### Baseline characteristics

A total of 7,646 patients were finally included in the present study, including 5,324 patients in the original cohort extracted from the MIMIC-IV database and 2,322 patients in the validation cohort extracted from an eICU-CRD database. The flow chart of the included population was shown in Fig. 1.

The included patients were divided into two groups according to the optimal cut-off value of PAR: 1,791 in the high-PAR group (≥ 7.2), and 3,533 in the low-PAR group (< 7.2). As exhibited in Table 1, compared with patients in the low-PAR group, patients in the high-PAR group have a lower proportion of male gender, liver diseases, HGB and bilirubin, a higher proportion of PPI usage, SOFA score, myocardial infarction, heart rate, RR, WBC, PLT, albumin, PAR, anion gap, glucose, potassium, INR, PT, stage II, stage III and pAKI.

**Table 1:** Comparisons of baseline characteristics in all cohorts

Covariates	Original cohort			Validation cohort		
	Low PAR	High PAR	P	Low PAR	High PAR	p
N	3533	1791	-	1489	833	-
Age, years	65.2 (15.9)	65.2 (16.0)	0.992	65.0 (15.5)	64.5 (14.9)	0.516
Gender, male, n (%)	2243 (63.5)	933 (52.1)	< 0.001	842 (56.5)	403 (48.4)	< 0.001
Weight, kg	27.8 (7.9)	28.0 (8.8)	0.688	30.4 (8.4)	28.6 (8.2)	0.318
Ethnicity, n (%)			0.167			0.579
White	2250 (63.7)	1186(66.2)		1182 (79.4)	671 (80.6)	
Black	481 (13.6)	234 (13.1)		170 (11.4)	96 (11.5)	
Other	802 (22.7)	371 (20.7)		25 (16.2)	19 (29.2)	
Interventions, n (%)						
MV use	2251 (63.7)	1130(63.1)	0.679	839 (56.3)	497 (59.7)	0.132
RRT use	386 (10.9)	216 (12.1)	0.234	119 (8.0)	81 (9.7)	0.177
Drugs usage, n (%)						
ACEI/ARB	1028 (29.1)	532 (29.7)	0.669	392 (26.3)	214 (25.7)	0.775
βblockers	2454 (69.5)	1278 (71.4)	0.162	916 (61.5)	506 (60.7)	0.747
CCB	882 (25.0)	431 (24.1)	0.493	324 (21.8)	188 (22.6)	0.690
Diuretic	2730 (77.3)	1368 (76.4)	0.488	1087 (73.0)	594 (71.3)	0.408
Statin	1527 (43.2)	770 (43.0)	0.897	606 (40.7)	340 (40.8)	0.991
Aspirin	1779 (50.4)	940 (52.5)	0.150	695 (46.7)	396 (47.5)	0.721
PPI	2292 (64.9)	1230 (68.7)	0.006	758 (50.9)	429 (51.5)	0.817
Score system, points						
SOFA	3.9 (1.1)	4.5 (1.6)	< 0.001	6.1 (3.6)	8.1 (3.1)	< 0.001
OASIS	38.4 (9.6)	38.2 (9.5)	0.435	29.8 (10.7)	30.4 (10.6)	0.141
APSIII	68.7 (27.6)	67.7 (26.7)	0.192	65.7 (25.1)	67.1 (25.2)	0.206
Comorbidities, n (%)						
Hypertension	1173 (33.2)	636 (35.5)	0.099	841 (56.5)	484 (58.1)	0.475
Diabetes	1220 (34.5)	662 (37.0)	0.085	554 (37.2)	343 (41.2)	0.066
CKD	1122 (31.8)	601 (33.6)	0.195	363 (24.4)	194 (23.3)	0.590
Myocardial infarct	670 (19.0)	386 (21.6)	0.028	149 (10.0)	65 (7.8)	0.092
CHF	1269 (35.9)	682 (38.1)	0.130	302 (20.3)	153 (18.4)	0.289
COPD	240 (6.8)	137 (7.6)	0.274	243 (16.3)	146 (17.5)	0.491
Liver disease	948 (26.8)	280 (15.6)	< 0.001	90 (6.0)	22 (2.6)	< 0.001
CCI, points	6.4 (2.8)	6.5 (3.2)	0.486	4.5 (1.8)	4.2 (1.6)	0.080
Vital signs						
MAP, mmHg	106.8 (30.3)	107.7 (31.1)	0.333	85.6 (25.0)	86.2 (24.0)	0.565
Heart rate, bpm	106.6 (21.8)	111.2 (24.1)	< 0.001	106.5 (29.1)	110.5 (27.2)	0.001
RR, bpm	28.8 (6.8)	29.7 (7.0)	< 0.001	25.6 (9.1)	26.1 (9.3)	0.205
Laboratory results						
WBC, × 10^9^/L	15.4 (5.7)	18.6 (6.9)	< 0.001	13.8 (5.4)	17.3 (8.3)	< 0.001
HGB, g/dL	9.6 (2.3)	9.4 (2.1)	0.001	10.9 (2.3)	10.5 (2.1)	< 0.001
PLT, × 10^9^/L	164.5 (67.6)	331.0(94.8)	< 0.001	150.0 (64.4)	311.9 (109.6)	< 0.001
HCT, %	34.6 (7.6)	34.5 (6.6)	0.819	33.0 (6.8)	32.3 (6.1)	0.010
Albumin, g/dL	3.9 (0.9)	3.0 (1.0)	< 0.001	3.5 (0.8)	2.9 (0.8)	< 0.001
PAR	4.3 (1.6)	12.0 (5.1)	< 0.001	54.3 (1.7)	11.4 (5.0)	< 0.001
Bilirubin, mmol/L	3.3 (1.9)	2.2 (1.4)	< 0.001	1.7 (0.9)	1.1 (0.7)	< 0.001
Anion gap, mEq/L	18.3 (5.4)	18.7 (5.8)	< 0.001	13.2 (5.9)	13.4 (6.2)	0.393
Bicarbonate, mEq/L	23.6 (4.6)	23.7 (4.7)	0.683	22.8 (5.9)	23.6 (6.4)	0.003
BUN, mg/dL	38.8 (7.5)	39.1 (10.3)	0.742	38.6 (8.1)	38.4 (10.7)	0.002
Glucose, mg/dL	186.9 (77.8)	202.1(95.0)	< 0.001	166.9 (80.0)	183.9 (86.9)	0.004
Lactate, mmol/L	3.5 (1.5)	3.5 (1.8)	0.473	2.9 (0.9)	2.5 (0.8)	< 0.001
Potassium, mmol/L	4.8 (0.9)	4.9 (1.0)	< 0.001	4.2 (0.7)	4.2 (0.7)	0.670
Sodium, mmol/L	139.8 (5.7)	139.6 (5.7)	0.263	137.6 (5.7)	137.4 (5.5)	0.247
Calcium, mg/dL	8.6 (1.7)	8.6 (1.1)	0.290	8.5 (1.1)	8.6 (1.1)	0.123
Chloride, mmol/L	105.8 (7.3)	105.6 (7.4)	0.252	101.4 (7.8)	100.3 (7.7)	0.002
PT, s	19.9 (5.4)	19.0 (6.8)	0.024	18.9 (8.4)	18.1 (7.0)	0.019
APTT, s	50.7 (13.2)	50.0 (14.6)	0.497	38.0 (10.5)	38.0 (10.9)	0.992
INR	1.9 (0.7)	1.8 (0.8)	0.010	1.7 (0.8)	1.6 (0.7)	0.009
AKI stage, n (%)			0.002			0.085
Stage I	2760 (78.1)	1322 (73.8)		1205 (80.9)	649 (77.9)	
Stage II	410 (11.6)	253 (14.1)		97 (6.5)	61 (7.3)	
Stage III	363 (10.3)	216 (12.1)		187 (12.6)	123 (14.8)	
Clinical outcome						
pAKI, n (%)	753 (21.3)	787 (43.9)	< 0.001	300 (20.1)	301 (36.1)	< 0.001

PAR, platelet-to-albumin ratio, MV, mechanical ventilation, RRT, renal replacement therapy, ACEI/ARB, Angiotensin converting enzyme inhibitors/Angiotensin receptor blockers, CCB, Calcium calcium blockers, NSAID, nonsteroidal anti-inflammatory drug, PPI, proton pump inhibitor, SOFA, sequential organ failure assessment, OASIS, oxford acute severity of illness score, APSIII, acute physiology score III, CKD, chronic kidney disease, CHF, congestive heart failure, COPD, chronic obstructive pulmonary disease, CCI, charlson comorbidity index, AKI, acute kidney injury, MAP, mean arterial pressure, RR, respiratory rate, WBC, white blood cell, HGB, hemoglobin, PLT, platelet, HCT, hematocrit, ALP, alkaline phosphatase, BUN, blood urea nitrogen, PT, prothrombin time, APTT, activated partial thromboplastin time, INR, international normalized ratio, AKI, acute kidney injury, pAKI, persistent AKI.

### Association of PAR with the outcome

A progressive increase in serum creatinine is closely associated with poor prognosis in AKI patients. Then, we analyzed the change before and after the diagnosis of pAKI and found that the mean serum creatinine measured on admission was 1.63 ± 0.94, and serum creatinine at first pAKI diagnosis was 2.40 ± 1.04, while the mean serum creatinine measured 48 h after pAKI diagnosis was 2.12 ± 1.02. A total of 4,131 (77.6%) patients showed a decrease in the serum creatinine 48 h after pAKI diagnosis, while 1,193 (22.4%) patients showed a positive change in the serum creatinine, reflecting a progressive increase in serum creatinine and a worsening kidney function in more than 20% diagnosed pAKI patients, and all results exhibited a similar tendency in the validation cohort (Table 2).

**Table 2:** Change in serum creatinine before and after the diagnosis of AKI

Variables	Values
Original cohort	
Serum creatinine at ICU admission, mean ± SD	1.63 ± 0.94
Serum creatinine at ICU admission, median (IQR)	1.00 (0.70, 1.80)
Serum creatinine at first AKI diagnosis, mean ± SD	2.40 ± 1.04
Serum creatinine at first AKI diagnosis, median (IQR)	1.70 (1.20–2.90)
Serum creatinine at 48 h after AKI diagnosis, mean ± SD	2.12 ± 1.02
Serum creatinine at 48 h after AKI diagnosis, median (IQR)	1.50 (0.93–2.68)
Positive changes of serum creatinine, n (%)	1193 (22.4)
Δserum creatinine, mean ± SD	0.86 ± 0.73
Negative or static change of serum creatinine, n (%)	4131 (77.6)
Δserum creatinine, mean ± SD	-0.63 ± 0.51
Validation cohort	
Serum creatinine at ICU admission, mean ± SD	1.53 ± 0.52
Serum creatinine at ICU admission, median (IQR)	1.08 (0.72, 1.90)
Serum creatinine at first AKI diagnosis, mean ± SD	2.74 ± 1.04
Serum creatinine at first AKI diagnosis, median (IQR)	2.10 (1.38, 3.49)
Serum creatinine at 48 h after AKI diagnosis, mean ± SD	2.12 ± 1.02
Serum creatinine at 48 h after AKI diagnosis, median (IQR)	1.50 (0.93, 2.68)
Positive changes of serum creatinine, n (%)	333 (14.3)
Δserum creatinine, mean ± SD	0.53 ± 0.42
Negative or static change of serum creatinine, n (%)	1989 (85.7)
Δserum creatinine, mean ± SD	-0.81 ± 0.67

ICU, intensive care unit, SD, standard deviation, IQR, interquartile range, AKI, acute kidney injury

The results of Cox proportional hazards regression were presented in Table 3. When adjusted for age, gender, weight, and ethnicity in Model I, the adjusted HR (95% CI) value of the high-PAR group was 2.89 (2.56–3.27, *P* < 0.001). When adjusted for model 1 plus comorbidities and the charlson comorbidity index in Model II, the adjusted HR value of the high-PAR group was still statistically significant (HR: 3.12, 95% CI: 2.74–3.54, *P* < 0.001). When further adjusted for model 2 plus score system, interventions, and drug usage, the adjusted HR value of the high-PAR group was still statistically significant (HR: 3.31, 95% CI: 2.91–3.78, *P* < 0.001). When further adjusted for model 3 plus vital signs and laboratory results except for platelets and serum albumin, the adjusted HR value of the high-PAR group was still statistically significant (HR: 3.45, 95% CI: 3.02–3.95, *P* < 0.001). We also performed this in the validation cohort, the results further confirmed that the high-PAR group was associated with pAKI (HR: 2.24, 95% CI: 1.86–2.71, *P* < 0.001). All these results suggested thatby using different models to control confounders, the results are solid that high PAR was positively associated with increased risk of pAKI.

**Table 3:** Summary of results of primary outcome and sensitivity analysis

	Original cohort		Validation cohort	
	HR (95%CI)	P value	HR (95%CI)	P value
Unadjusted	2.89 (2.56–3.27)	< 0.001	2.24 (1.86–2.71)	< 0.001
Model 1	2.93 (2.59–3.32)	< 0.001	2.30 (1.90–2.79)	< 0.001
Model 2	3.12 (2.74–3.54)	< 0.001	2.36 (1.94–2.87)	< 0.001
Model 3	3.31 (2.91–3.78)	< 0.001	2.55 (2.05–3.16)	< 0.001
Model 4	3.45 (3.02–3.95)	< 0.001	2.77 (2.13–3.60)	< 0.001

Model 1 adjusted for age, gender, weight, ethnicity. Model 2 adjusted for model 1 plus comorbidities and charlson comorbidity index. Model 3 adjusted for model 2 plus score systerm, interventions, and drug usage. Model 4 adjusted for model 3 plus vital signs and laboratory results except for platelets and serum albumin

### The predictive role and superiority of PAR in predicting pAKI in patients admitted to ICU

**Fig. 2:**
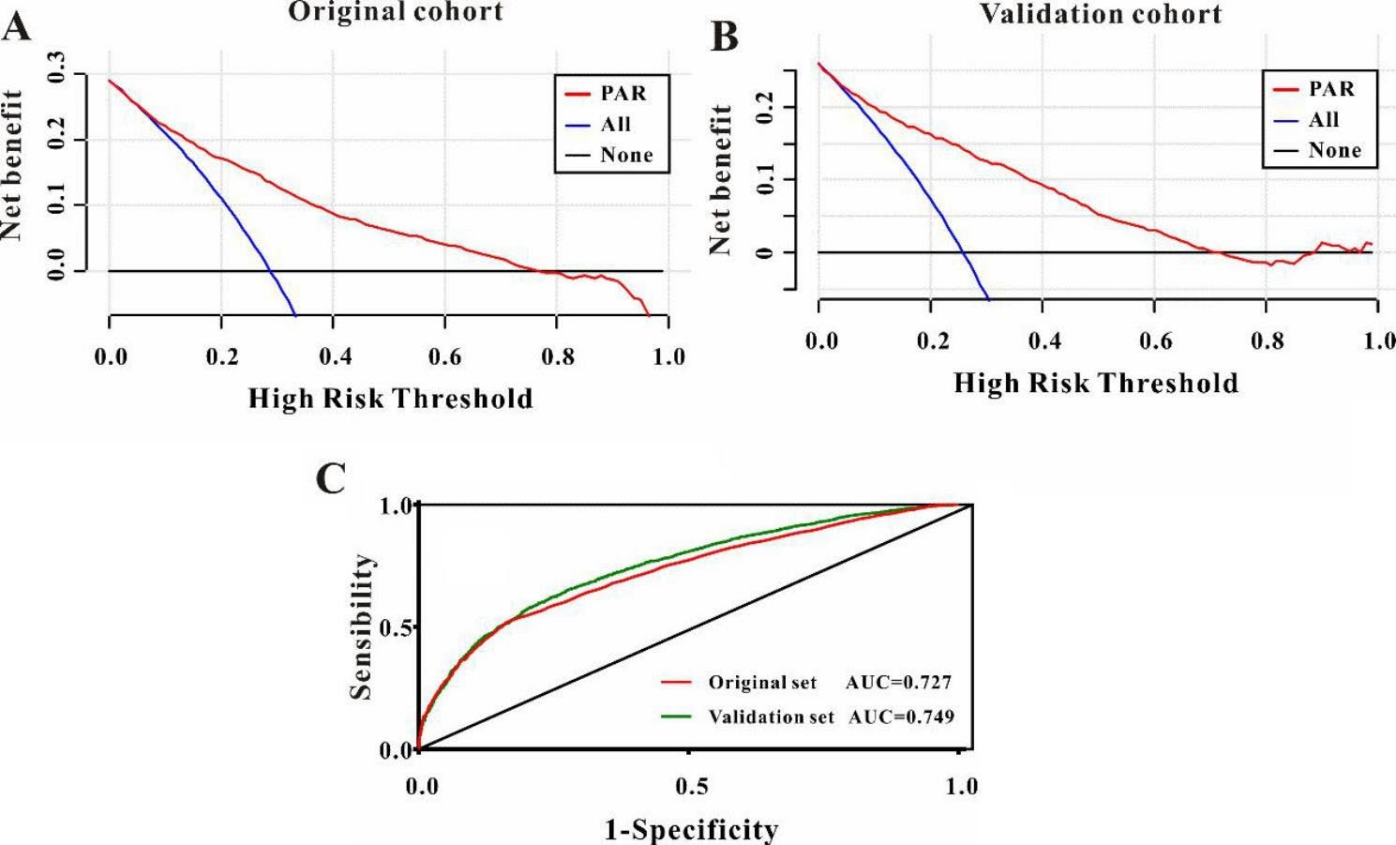
Decision curves analysis of PAR values for predicting the persistent acute kidney injury in **(A)** the original cohort and **(B)** the validation cohort and **(C)** receiver operating characteristic curve analysis of PAR for persistent acute kidney injury in the original cohort, and in the validation cohort

Then, decision curve analysis (DCA) was performed to determine the clinical utilities of the PAR in predicting pAKI in patients admitted to ICU. The results indicated that the PAR was clinically useful for predicting pAKI in patients admitted to ICU in the original cohort as well as in the validation cohort (Fig. 2A-B). When predicting the pAKI for patients admitted to ICU, the PAR exhibited good predictive abilities in the original cohort (C-index: 0.726, 95%CI: 0.714–0.739, Fig. 2C) and in the validation cohort (C-index: 0.744, 95%CI:0.722–0.766, Fig. 2C).

As an unexplored systemic inflammatory biomarker in predicting pAKI, we intended to compare the predictive abilities between NLR, PLR, MLR, SII, and PAR by conducting the ROC curve analysis. As shown in Table 4, the AUC of PAR was greater compared to the AUC of NLR, PLR, MLR, and SII both in the original cohort and the validation cohort (*P* < 0.01, respectively, Table 4). Consequently, these data depicted that the novel systemic inflammatory response biomarker of PAR was superior to other systemic inflammatory response biomarkers (NLR, PLR, MLR, and SII) when predicting the pAKI for patients admitted to ICU.

**Table 4:** Receiver operating curve analysis

Variable	Sensitivity	Specificity	AUC (95%CI)	P value
Original cohort				
PAR	60.3	74.3	0.726 (0.714–0.739)	
NLR	12.3	90.8	0.504 (0.486–0.519)	< 0.001
PLR	37.5	76.6	0.603 (0.589–0.616)	< 0.001
MLR	28.2	74.3	0.501 (0.488–0.515)	< 0.001
SII	43.4	79.5	0.615 (0.601–0.628)	0.004
Validation cohort				
PAR	70.7	69.7	0.744 (0.722–0.766)	
NLR	48.3	56.4	0.507 (0.485–0.529)	< 0.001
PLR	80.4	28.6	0.602 (0.580–0.624)	< 0.001
MLR	54.0	48.6	0.503 (0.481–0.525)	< 0.001
SII	49.3	62.2	0.507 (0.485–0.529)	< 0.001

PAR, platelet to serum albumin ratio, NLR, neutrophil-lymphocyte ratio, PLR, platelet-lymphocyte ratio, MLR, monocyte-lymphocyte ratio, SII, systemic immune-inflammation index, area under the receiver operating curve

## Discussion

The results in the present studyconfirmed that higher PAR on admission was significantly associated with an increased risk of pAKI in patients admitted to ICU and a PAR cut-off of 7.2 that provided excellent discriminative properties for early risk stratification of pAKI in ICU patients. Furthermore, PAR tended to be a better predictor for pAKI in patients admitted to ICU compared with NLR, PLR, MLR, and SII.

AKI is a serious complication for critically ill patient and these patients bear a bad prognosis especially when it needs RRT. Recently, numerous studies focused on the duration of AKI as an important component affecting clinical outcomes in different disease conditions [9]. According to previously reported, worse long-term outcomes, including ESRD and significantly reduced survival, occurred in patients with pAKI compared to patients without AKI or transient AKI [21, 22]. Perinel et al. demonstrated that pAKI developed in 39% of ICU patients (175 of 447) and that it was associated with higher in-hospital mortality (38.9%) compared with transient AKI (29.6%) and no AKI (23.8%) [11]. Roman-Pognuz et al. investigated the incidence of pAKI in cardiac arrest patients and suggested that pAKI occurs in more than one third and pAKI is associated both with survival and with the length of stay at the hospital [23]. In the present study, 1193 (22.4%) patients showed a positive change in the serum creatinine 48 h after AKI diagnosis, reflecting a worsened kidney function in pAKI patients. It should be noted that, although a small percentage of pAKI patients progress to severe AKI or needed to receive RRT, early recognition and risk stratification of pAKI is important for preventing or minimizing the associated adverse outcomes [24].

A large number of investigators paid attention to seek biomarkers to predict pAKI. Roman-Pognuz et al. demonstrated that high doses of adrenaline, serum lactate levels, and dobutamine could predict pAKI in patients who survive cardiac arrest [23]. Lumlertgul et al. aimed to explore whether urine neutrophil gelatinase-associated lipocalin (uNGAL) can predict pAKI and major adverse kidney events in AKI patients and found that uNGAL can accurately predict pAKI [25]. Moreover, Qiu et al. analyzed 90 patients in critically ill patients with sepsis and indicated that serum hepcidin levels measured when AKI was diagnosed exhibited good predictive value to predict the occurrence of persistent AKI in septic patients admitted to ICU [26]. In the present study, we intended to test an easy to perform and present low cost and high analytical sensitivity prognostic biomarker based on the routine laboratory tests, PAR in the predictive of pAKI in patients admitted to ICU. Our results demonstrated that PAR on admission was markedly linked to an increased risk of pAKI in patients admitted to ICU.

However, the mechanisms to explain the association between PAR and pAKI have not been fully understood. Higher PAR, which means higher platelets counts with inflammation, low albumin levels with poor nutrition, which has been identified as a novel indicator and potential prognostic biomarker that can reflect the systemic inflammation and immune nutrition status, can predict a poor prognosis in various conditions [27]. Platelets can also trigger and exacerbate inflammation through interaction with a variety of immune cells and secretion of pro-inflammatory cytokines, and inflammation drives the development of malnutrition, which may in turn amplify systemic inflammatory responses, leading to a vicious cycle [28]. Inflammation is broadly recognized as an important factor in the pathogenesis of AKI, and AKI is now considered a kidney-centered inflammatory syndrome, inflammatory responses, including innate and adaptive immune responses, are involved in the initiation and development of acute kidney injury, increased inflammatory factors increased risk of AKI in critically ill patients [29, 30]. Moreover, malnutrition is extremely common in ICU patients, as the majority of patients in ICU have either a serious illness, trauma, or have had major surgery and are therefore unable to maintain their own nutritional needs [31]. Furthermore, our data verified that PAR was superior to other systemic inflammatory response biomarkers (NLR, PLR, MLR, and SII) when predicting the pAKI for patients admitted to ICU. Taken together, higher PAR could predict pAKI may be due to the activation of the systemic inflammatory response and malnutrition in patients admitted to ICU. The precise mechanism still needs to be clarified in the future.

Several limitations should be mentioned in this study. First of all, the present study was a retrospective study based on two public databases, and the results should be further verified by future prospective studies or randomized controlled studies. Moreover, the pAKI was according to KDIGO-AKI criteria based on serum creatinine in the first 48 h of their ICU admission [18], there were many cases of patients with oliguria may be excluded from this study, hence, this will cause bias to the results, our results should be further confirmed by more studies in the future.

## Conclusions

This study provided evidence that higher PAR on admission was significantly linked to an increased risk of pAKI in patients admitted to the ICU. As a low-cost, simple, and promising prognostic marker, PAR exhibited good predictive ability for the risk stratification of pAKI in ICU patients.

## Electronic supplementary material

Below is the link to the electronic supplementary material.

**Additional file 1.** The raw data used in this manuscript extracted from eICU-CRD database.

**Additional file 2.** The raw data used in this manuscript extracted from MIMIC-IV database.

## Data Availability

All data generated or analysed during this study are included in this published article [and its supplementary information files].

## Declarations

**Ethics approval and consent to participate**.

The datasets used for the current study come from eICU-CRD and MIMIC-IV. The study was conducted in accordance with the Declaration of Helsinki (as revised in 2013). This project was both approved by Beth Israel Deaconess Medical Center (BIDMC) and the institutional review boards of Massachusetts Institute of Technology (MIT) (certification number: 9322422) and individual consent for this retrospective analysis was waived by the BIDMC and the institutional review boards of Massachusetts Institute of Technology (MIT).

**Consent for publication**.
